# Author Correction: Forensic characterization of 15 autosomal STRs in four populations from Xinjiang, China, and genetic relationships with neighboring populations

**DOI:** 10.1038/s41598-022-15910-3

**Published:** 2022-07-13

**Authors:** Xiaoni Zhan, Atif Adnan, Yuzhang Zhou, Amjad Khan, Kadirya Kasim, Dennis McNevin

**Affiliations:** 1 Department of Forensic Genetics, School of Forensic Medicine, China Medical University, Shenyang, 110122 People’s Republic of China; 2 Faculty of Science & Technology, National Centre for Forensic Studies, University of Canberra, Canberra, Australia

Correction to: *Scientific Reports* 10.1038/s41598-018-22975-6, published online 16 March 2018

After publication of this Article it transpired that the Authors did not secure consent to publish the microsatellite genotype data of the participants. The Supplementary Information file containing anonymised genetic data was therefore removed. As a result, all in-text citations linked to the Supplementary Information file have been corrected or removed. The anonymised data will be available to readers who may wish to access it from Dennis McNevin, provided appropriate clearance is secured from the original ethics committee.

